# Developmental differences in the visual processing of emotionally ambiguous neutral faces based on perceived valence

**DOI:** 10.1371/journal.pone.0256109

**Published:** 2021-08-16

**Authors:** Leslie Rollins, Erin Bertero, Laurie Hunter

**Affiliations:** 1 Department of Psychology, Christopher Newport University, Newport News, VA, United States of America; 2 Eastern Virginia Medical School, Norfolk, VA, United States of America; Bournemouth University, UNITED KINGDOM

## Abstract

The aim of the present research was to assess age-related differences in how children and adults interpret and visually process emotionally ambiguous neutral faces. Children and adults provided neutral child faces with valence and arousal ratings while eye movements were recorded. Consistent with previous research, children and adults both interpreted the neutral faces as negatively valenced. Both age groups showed fewer fixations to the eye region when they rated the faces as positive. However, adults fixated more on the eye region when they rated the faces as negative whereas children fixated more on the eye region when they rated the faces as neutral. This finding may suggest that children strategically allocate attentional resources to the eye region when processing emotionally ambiguous faces to gather critical affective information. These findings have implications for the utilization of neutral faces as an experimental control condition and serve as the foundation for future research on the development of mechanisms that underlie the interpretation of emotionally ambiguous faces.

## Introduction

Facial expressions are considered to be the richest and most descriptive sources of emotional expression. Ekman [1], building on Darwin’s [2] work on the physiology of facial expressions, identified muscular movements associated with each of the six discrete, basic emotions (i.e., anger, happiness, surprise, disgust, sadness, and fear). For instance, a curled lip conveys anger whereas a smile conveys happiness [1]. Consistent with the framework provided by Ekman, studies exploring facial expressions of emotion typically use neutral faces as a control. Neutral faces are characterized by the relaxation of the facial muscles, and it is assumed they hold no emotional value [3, 4]. More recent research, however, has suggested this assumption may not be valid.

Rather than being perceived as neutral, neutral faces are emotionally ambiguous and typically perceived with a negativity bias. Neutral faces are associated with less accurate and slower emotion recognition judgments [5, 6]. For example, Kesler-West et al. [6] observed that emotion labeling was less accurate for neutral faces than happy, sad, angry, and frightened faces. In support of a negativity bias, neutral faces were more often misclassified as sad, angry, or frightened than happy [6]. Further support for neutral faces being processed with a negativity bias come from Go/No-Go [7], implicit affective association [8], and visual search tasks [9]. For example, Park et al. [9] demonstrated individuals to be slower and less accurate on a visual search task when neutral faces were presented among frowning faces than smiling faces as well as less accurate at detecting an expression change was when the expression was changed from neutral to frowning (or vice versa) relative to neutral or frowning to smiling (or vice versa).

To account for emotionally ambiguous faces being processed with a negativity bias, some researchers have proposed the initial-negativity hypothesis. According to the initial-negativity hypothesis, there is an initial bias toward processing emotionally ambiguous faces, such as those displaying neutral and surprised expressions, as negatively valenced [10]. However, that response can be overridden by emotion regulation [10]. In support of the initial-negativity hypothesis, Neta et al. [11] observed that initial visual fixations determine whether ambiguous surprised facial expressions are interpreted as negative or positive. Another study showed surprised facial expressions are rated more positively when reaction times are delayed, suggesting that effortful emotion regulation processes support the reinterpretation of ambiguous facial expressions [12].

One implication of the initial-negativity hypothesis is that children may interpret emotionally ambiguous facial expressions as more negative than adolescents and adults due to immature emotion regulation abilities [13]. Relatively few developmental studies to date have focused on valence biases in emotion recognition for emotionally ambiguous stimuli. However, Tottenham et al. [14] showed that children and early adolescents exhibited a negativity bias while rating the valence of surprised facial expressions, whereas older adolescents rated them ambivalently. Further, there was a trend toward older adolescents taking longer to rate the expression of neutral faces than younger children [14]. This finding suggests that younger children may have quickly rated them as negative, whereas older adolescents may have taken longer due to the perception of the faces as emotionally ambiguous. Psychophysiological data provides additional evidence in favor of children displaying a larger negativity bias for emotionally ambiguous faces than older adolescents. Tottenham and colleagues [14] also assessed behavioral ratings and the activation of the corrugator supercilii muscle, which draws the eyebrows together during negative expressions, while 6- to 9-year-old children, 10- to 13-year-old young adolescents, and older 14- to 17-year-old adolescents viewed fearful, angry, happy, neutral, and surprised faces. Relative to older adolescents, younger children showed stronger corrugator activity to emotionally ambiguous neural and surprised faces. These findings suggest children and young adolescents may process emotionally ambiguous faces similar to negatively valenced faces and this negative valence bias reduces during adolescence, perhaps coinciding with the development of emotion regulation.

In summary, emotionally ambiguous faces are processed with a negativity bias and the initial-negativity hypothesis suggests that there should be age-related changes in the processing of emotionally ambiguous faces due to the development of emotion regulation. Although previous research has examined how eye movements are associated with the interpretation of emotionally ambiguous surprise faces [11], to our knowledge, similar research has not been conducted with neutral faces. Therefore, the current study examined valence ratings and eye movements to emotionally ambiguous neutral faces in children and adults. We expected to observe a negativity bias in valence ratings and explored whether the negativity bias was larger in children than adults [cf. 14]. Further, based on the results by Tottenham et al. [14] and Neta et al. [11], we anticipated observing age-related differences in eye movements based on whether faces were rated as negative, neutral, or positive.

## Method

### Participants

Thirty child participants (15 girls, 15 boys; mean age = 9.47 years, *SD* = 1.53, range = 7.29–11.93 years; 8 Black, 1 Black/White, 21 White) were recruited from the local community and received a small toy in compensation for their time. Thirty adult participants (23 women, 7 men; mean age = 19.9, *SD* = 1.08, range = 17.95–22.01 years; 4 Black, 1 Asian/White, 25 White) were recruited from the University’s undergraduate participant pool and received course credit in compensation for their time. Exclusion criteria included a history of a neurological or behavioral disorder (e.g., autism, ADHD/ADD, history of traumatic brain injury).

### Face stimuli

Two sets of 40 stimuli (872 x 872 pixels) were selected from the Child Affective Facial Expression (CAFE) database, a validated stimulus set of approximately 1200 photographs of 2- to 8-year-old children [15]. All faces showed front-view faces with a neutral, closed mouth expression. Stimuli were selected to maximize racial and ethnic diversity as much as possible given the faces available within the stimulus set. Within each sex and ethnic group, stimuli were randomized into two face sets. Face sets were counterbalanced across participants and matched for sex and race/ethnicity. Four additional neutral faces, one happy face, and one sad face were used for the instructional phase of the task.

### Procedure

The University’s Institutional Review Board approved all procedures prior to data collection. Participants were seated in a stationary chair at a distance of 60 cm from a 24-inch monitor that was equipped with a Tobii X3-120 eye tracking system (Tobii Technology, Danderyd, Sweden) underneath. The eye-tracker noninvasively record participant gaze using corneal reflection at a sampling frequency of 120 Hz. Participants were instructed to remain as still as possible during data collection. Tobii Studio Pro software (Tobii Technology, Danderyd, Sweden) was used for stimulus presentation as well as collection of eye-tracking and behavioral data. Prior to beginning the task, calibration was performed using a standard 5-point calibration procedure. The administrator sat with the participants throughout data collection to answer questions in case they arose and to ensure correct task completion.

During the instructional phase, participants were told they would see faces and then rate them for pleasantness and intensity on a 7-point scale once they were removed from the screen. Consistent with Russell’s circumplex model of emotions [16], pleasantness was rated -3 to +3, ranging from “Very Negative” to “Very Positive” and intensity was rated -3 to +3, ranging from “Very tired” to “Very energetic.” Theoretically, neutral facial expressions would be identified with a score of 0 for pleasantness and arousal. Participants completed a brief practice phase to ensure full comprehension of the instructions. The practice paradigm included three pairs of faces (i.e., a happy versus neutral face, a sad versus neutral face, and two neutral faces) to highlight rating differences and the range of the scales provided. For the test phase, eye tracking data was recorded as participants viewed each face for 4 seconds. Then, once the face was removed from the screen, participants rated the face for pleasantness and intensity with a keyboard press.

### Data processing

Prior to data collection, seven areas of interest (AOIs) were defined for each face using Tobii Studio Pro software: whole picture (i.e., rectangle), face (i.e., oval excluding hair and ears), eyes (combined across the left and right eye for analysis), nose, mouth, forehead, and cheeks (combined across the left and right eye for analysis). The AOIs were defined based on features important for emotion recognition and AOIs used in previous research [e.g., 17, 18]. Fixation duration and fixation count metrics were exported for each stimulus and AOI from Tobii Studio Pro. A fixation was a series of data points in which the eyes did not shift more than 35 pixels for at least 60 ms. Average fixation counts and durations were calculated as a function of perceived valence (i.e., negative, neutral, positive). Faces perceived as negative were those given a valence rating of -1 to -3, faces perceived as neutral were those given a rating of 0, and faces perceived as positive were those given a rating of 1 to 3. The number of trials for each perceived valence category are provided in Table 1. A minimum of 4 trials per valence rating were required for inclusion in the analyses of eye movements. This resulted in the exclusion of five children and three adults for the eye-tracking analyses. Due to the similarity of the results, only analyses on fixation count are reported.

**Table 1:** Descriptive statistics for the number of trials in each perceived valence category.

		Mean	Minimum	Maximum
Children	Negative	16	7	29
	Neutral	14	4	24
	Positive	10	4	28
Adults	Negative	17	6	24
	Neutral	13	7	23
	Positive	10	4	22

## Results

### Behavioral data

**Fig 1:**
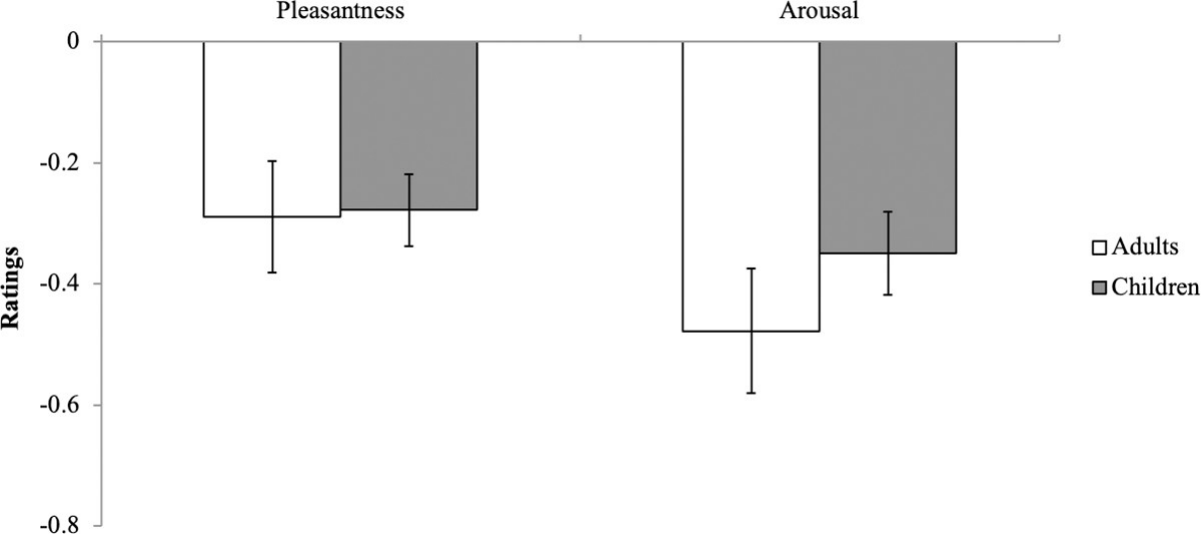
Pleasantness and arousal ratings for neutral child faces. A score of zero coincides with a neutral perception of the face.

No significant differences between age groups were found for interpretation of the emotional expression of the faces. Independent-sample *t*-tests showed children and adults provided the faces with similar pleasantness, *t*(58) = 0.098, *p* = 0.922, and intensity, *t*(58) = 1.038, *p* = 0.304, ratings (see Fig 1). Additionally, one-sample *t*-tests comparing pleasantness and arousal valence scores relative to a neutral rating of 0 demonstrated that both children, *t*(29) = -3.125, *p* = .004, and young adults, *t*(29) = -4.669, *p* < .001, judged the faces as unpleasant. Similarly, both children, *t*(29) = -4.635, *p* < .001, and adults, *t*(29) = -5.109, *p* < .001, judged the faces as and low on intensity, *t*(59) = -6.682, *p* < 0.001.

### Eye-tracking data

A 2 Age Group (Children, Adults) x 3 Perceived Valence (Positive, Negative, Neutral) x 5 Feature (Eyes, Forehead, Mouth, Nose, Cheek) omnibus ANOVA was calculated on fixation counts. The analysis revealed a significant main effect of feature, *F*(4, 192) = 131.365, *p* < .001. Participants showed the most fixations to the eye region, an intermediate number of fixations to the nose and forehead regions, and the fewest fixations to the mouth and cheek regions. The main effect of feature was also qualified by Feature x Age Group, *F*(4, 192) = 3.779, *p* = .006, Feature x Valence, *F*(8, 384) = 5.181, *p* < .001, and Feature x Perceived Valence x Age Group interactions, *F*(8, 384) = 3.121, *p* = .008. The main effect of Perceived Valence, the main effect of Age Group, and the Perceived Valence x Age Group interaction were not significant, *ps* ≥ .145.

**Fig 2:**
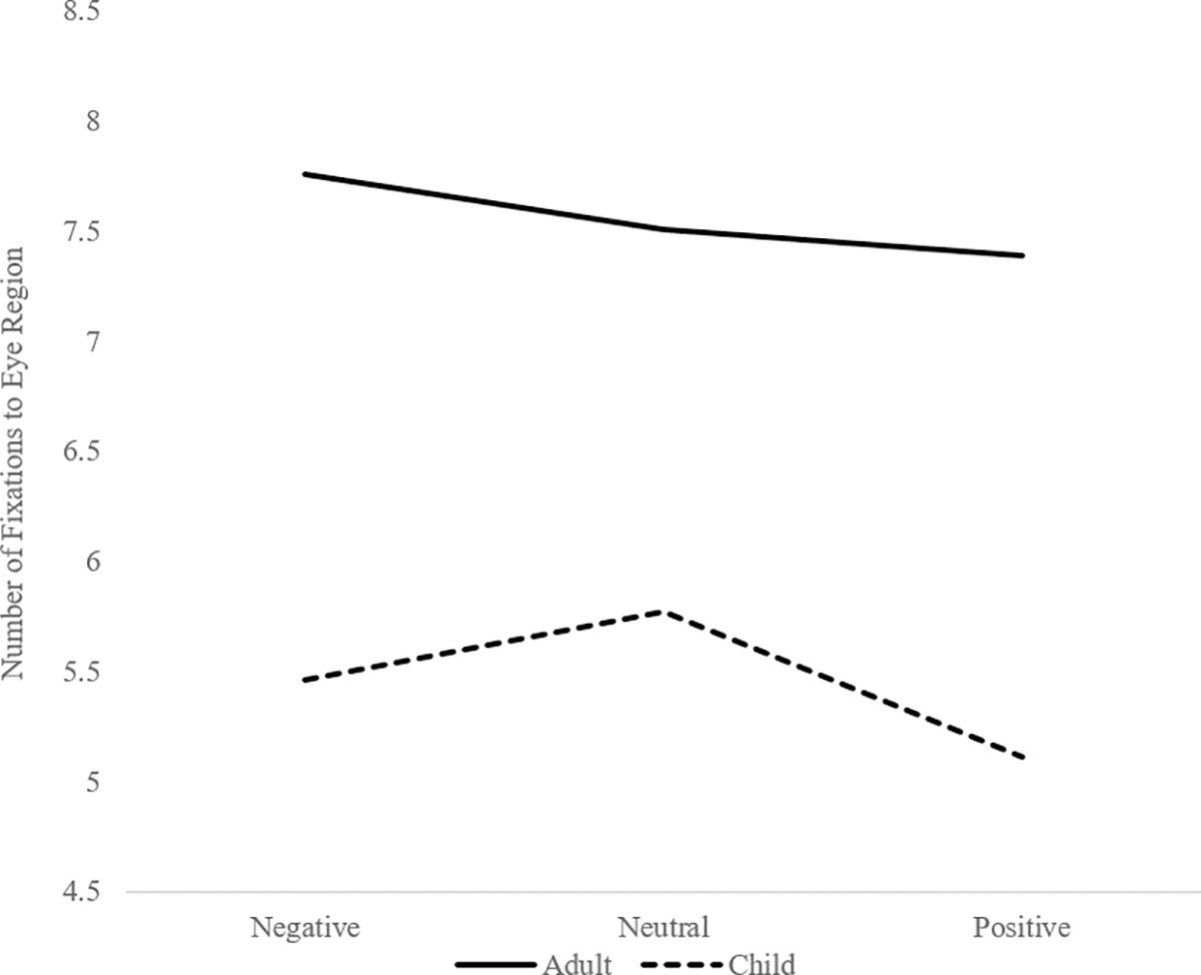
Fixations to the eye region of the face as a function of the perceived valence.

To assess the three-way interaction, follow-up analyses were conducted by performing 3 Perceived Valence x 2 Age Group ANOVAs separately for fixation counts to each facial feature. The main effect of Valence, the main effect of Age Group, and the Perceived Valence x Age Group interaction were not significant for the forehead, mouth, nose, and cheek regions, *ps* ≥ .069. However, for the eye region, the main effect of Perceived Valence, *F*(2, 100) = 6.43, *p* = .002, the main effect of Age Group, *F*(1, 50) = 8.66, *p* = .005, and the Perceived Valence x Age Group interaction, *F*(2, 100) = 3.47, *p* = .035, were all significant. Thus, separate ANOVAs assessing fixations to the eye region as a function of perceived valence were conducted for adults and children. Eye movements were marginally influenced by perceived valence in adults, *F*(2, 52) = 2.644, *p* = .081 (see Fig 2). Bonferroni post-hoc comparisons showed that adults fixated marginally more on the eye region of the faces they rated as negative relative to faces they rated as positive (*p* = .051); fixations to neutral faces did not differ from those to either positive (*p* = 1.0) or negative faces (*p* = .301). Eye movements in children varied significantly as a function of perceived valence, *F*(2, 48) = 6.845, *p* = .002 (see Fig 2). In contrast to the pattern exhibited by adults, children fixated more on the eyes of faces they rated as neutral relative to faces they rated as positive (*p* = .004); fixations to negative faces did not differ from those to neutral (*p* = .199) or positive faces (*p* = .234).

## Discussion

The aim of the current study was to assess age-related differences in the affective evaluation and perceptual processing of emotionally ambiguous neutral faces. Children and adults both rated emotionally ambiguous faces as negatively valenced, and there was no difference in the magnitude of the negativity bias between children and adults. To explore perceptual processing of emotionally ambiguous neutral faces, fixation counts were assessed as a function of participant age and whether the faces were identified as negative, neutral, or positive. Both age groups fixated less on the eye region of faces they rated as positive. Adults fixated more on the eye region of the faces they rated as negative relative to faces they rated as positive with fixations to neutral faces in between. In contrast, children fixated more on the eyes of faces they rated as neutral relative to faces they rated as positive with fixations to negative faces in between.

Rather than being perceived as neutral, faces designed to display a neutral expression were rated as negatively valenced. The observation of a negativity bias in valence ratings is consistent with findings from a variety of experimental paradigms [7–9, 14]. Taken together, these data suggest that it is problematic to assume that neutral faces hold no emotional value [3, 4]. Further, these findings have important implications for the utilization of neutral faces as a control condition for research on emotional face processing.

The magnitude of the negativity bias was comparable in 7- to 11-year-old children and adults. Tottenham et al. [14] similarly observed no age-related difference in the ratings of emotionally ambiguous neutral faces in 6- to 9-year-old children, 10- to 13-year-old young adolescents, and 14- to 17-year-old older adolescents. These data are relatively surprising when considered along with the initial-negativity hypothesis, which proposes that initial assessments of negativity can be overridden by emotion regulation. Because emotion regulation develops throughout childhood [e.g., 13], younger children would be expected to rate emotionally ambiguous facial expressions as more negative than older children and adolescents. One possibility is that this pattern may not have been observed in the current study or in the research conducted by Tottenham et al. [14] because responses were self paced, although Tottenham et al. [14] prompted their participants to respond as quickly as possible. Age-related differences in the negativity bias may emerge under speeded conditions, which limit the availability of controlled processes, or in younger samples of children.

Although children and adults provided emotionally ambiguous neutral faces with comparable valence ratings, they showed differences in how they perceptually processed the faces based on the valence ratings they provided. Adults showed the highest fixation counts to the eye region of the face when they rated the face as negative relative to positive, with neutral in between. This finding is consistent with research indicating preferential viewing of the eye region for negative facial expressions relative to positive facial expressions. Blais et al. [19] observed that the perceptual processing of the eye region was more diagnostic for the recognition of anger relative to other emotions. Further, numerous studies have shown that the eye region is initially fixated on and viewed longer for negative facial expressions, especially sadness and anger, relative to positive facial expressions [e.g., 20–22].

Similar to the adults, children showed the lowest fixation counts to the eye region of neutral faces they evaluated as positive. This finding is consistent with research in adults showing that the eye region is fixated less for positive facial expressions than negative facial expressions [e.g., 20–22]. However, unlike adults, children showed the highest fixation counts to the eye region of the face when they rated the face as neutral relative to positive, with negative in between. One explanation may lie in the cognitive allocation strategy used by children. If children perceive emotionally ambiguous facial expressions to be more difficult to interpret, they may draw their attention to the eyes due to the diagnostic information about emotional expression provided by this facial feature. Consistent with this notion, Tottenham et al. [14] found that older adolescents took longer to rate the emotion of neutral faces than younger adolescents and 6- to 9-year-old children. This finding could suggest that, although valence ratings were similar across groups, older adolescents perceived the neutral faces to be more emotionally ambiguous and delayed their response times to allow for additional strategic processing. Although this explanation is speculative, our current results provide evidence in favor of emotionally ambiguous faces being more demanding for children to judge. These findings can also be informed by motivational relevance theory [23]. Motivational relevance theory argues that attentional prioritization is determined by multiple factors, including the ambiguity and salience of the stimulus, task demands, and cognitive and emotional states of the individual [23]. As noted by Maratos and Pessoa [23], individuals should be motivated to attend to ambiguous stimuli because attention is necessary to determine relevance. Children should show greater preferential processing of emotionally ambiguous stimuli if they find their interpretation to be more difficult than older adolescents and adults.

A novel contribution of the present study to the current literature on the perceptual processing of facial expressions is that eye movements were examined as a function of the valence rating provided by participants, rather than the categorical emotional expression of the face. Our findings suggest that individual differences in valence ratings within an emotional expression may coincide with the perceptual processing of the face. The current study has some limitations that restrict the interpretations of the data. One limitation is that the stimulus set only included child faces. The physiognomic features of child faces may have influenced emotion ratings. Lorenz [24] argued that infant facial features (e.g., a round face and high forehead) are designed to promote positive affect and caregiving responses. Consistent with this claim, one study showed that young adults rated infant faces with a round face and a high forehead as cuter than infant faces with a narrow face and low forehead [25]. However, Luo et al. [26] showed that this effect dissipates by 4.5 years of age, which is younger than the faces used for the present study. The use of child faces could also impact the findings by facilitating children’s performance; a recent study of adolescents revealed an own-age bias in emotion recognition [27]. Another potential limitation is the inclusion of only emotionally ambiguous neutral faces. This could have led individuals to use a wider range of the valence scale than if neutral faces had been presented along with faces of other emotional expressions. Future research could assess perceptual processing strategies used while individuals process other emotionally ambiguous facial expressions. For example, Wiggins et al. [28] parametrically varied the emotional intensity of angry, fearful, and happy faces by morphing them with neutral faces at 50%, 75%, and 100% intensity. Emotion classification accuracy was lowest for angry and fearful faces at 50% intensity. Assessing whether individuals allocate more fixations to the emotionally ambiguous faces would be a good test of the hypothesis that children in the current study strategically allocated attention toward the eyes due to the diagnostic information about emotional expression provided by this facial feature. This approach is also important to examine developmentally because adolescents rate low intensity faces as more perceptually ambiguous than adults [29] and show different patterns of neural activation as a function of emotional intensity [28, 29].

In conclusion, the present study observed that children and adults provided emotionally ambiguous neutral faces with similar valence and arousal ratings. However, they showed different patterns of eye movements to the eye region of emotionally ambiguous neutral faces based on whether they judged them to be negative, neutral, or positive. Both age groups fixated less on the eye region when they rated the faces as positive. However, adults fixated more on the eye region when they rated the faces as negative and children fixated more on the eye region when they rated the faces as neutral. This finding may suggest that children allocate attentional resources to the eye region when processing emotionally ambiguous faces to gather critical affective information. Future research is needed to continue probing how the recognition of emotionally ambiguous expressions develops and the mechanisms that underlie this change throughout the lifespan.
